# Letter to the Editor Regarding Quantifying the Carbon Footprint of External Beam Radiation Therapy—A Narrative Review

**DOI:** 10.1002/jmrs.70028

**Published:** 2025-10-10

**Authors:** Ana Luísa Soares, Isabel Bravo, José Guilherme Couto

**Affiliations:** ^1^ Medical Physics Service Portuguese Oncology Institute of Porto Porto Portugal; ^2^ Medical Physics, Radiobiology and Radiation Protection Group, IPO Porto Research Center CI‐IPOP Portuguese Oncology Institute of Porto (IPO Porto)/Porto Comprehensive Cancer Centre (Porto.CCC) & RISE@CI‐IPOP (Health Research Network) Porto Portugal; ^3^ Radiography Department, Faculty of Health Sciences University of Malta Msida Malta

## Abstract

This letter highlights the valuable contribution of the review titled ‘Quantifying the Carbon Footprint of External Beam Radiation Therapy—A Narrative Review’ (https://doi.org/10.1002/jmrs.70009) in understanding how radiation therapy impacts healthcare‐related greenhouse gas emissions, particularly through pre‐treatment imaging, treatment delivery, and patient travel. Therefore, the letter emphasizes that empowering radiation therapists with green skills is essential to foster a culture of environmental responsibility within radiation therapy departments.

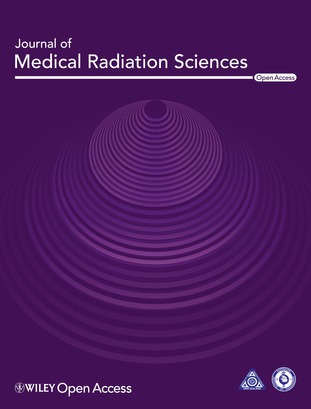

Kyeyune KM, Leech M. Quantifying the Carbon Footprint of External Beam Radiation Therapy—A Narrative Review. *J Med Radiat Sci*. https://doi.org/10.1002/jmrs.70009

We want to congratulate Kyeyune and Leech on their timely and insightful article, “Quantifying the Carbon Footprint of External Beam Radiation Therapy—A Narrative Review” [1]. Their work makes a valuable contribution to understanding how radiation therapy impacts healthcare‐related greenhouse gas emissions, particularly through pre‐treatment imaging, treatment delivery, and patient travel, and provides a solid foundation for further research in this area.

As indicated in Kyeyune and Leech's paper, in the context of the current climate emergency, it is critical that all healthcare sectors meaningfully engage with sustainability in their daily practice, including radiation therapy. The authors summarize the evidence and support the work of future researchers exploring this topic.

Their work closely aligns with broader concerns about integrating sustainable practices across all healthcare sectors. Soares et al. [2] previously identified nine key dimensions for the applicability of green practices within healthcare facilities (energy, water, procurement, building, food, travel, waste, behaviour, and green team). Kyeyune and Leech brilliantly summarise the evidence across two of these areas: energy and travel. According to Chuter et al. [3] these are the two most significant contributors to the carbon footprint of radiation therapy departments. However, we would like to encourage researchers worldwide to also explore the other dimensions, as they are significantly under‐researched. As demonstrated in the paper by Anudjo et al. [4] evidence of research exists only in some of the dimensions of sustainability.

Practices are still inconsistently implemented worldwide despite increasing awareness and urgency. Their review emphasizes the importance of integrating sustainability not only into infrastructure but also into clinical practice, workforce culture, and professional competencies [2].

Furthermore, the role of radiation therapists (RTs) in leading this transformation is crucial. As healthcare professionals, RTs are uniquely positioned to promote sustainability initiatives, as they typically treat patients five times a week. Moreover, when provided with the resources and knowledge to encourage and educate colleagues and radiation therapy patients, such as through supporting treatments in centres closer to patients' homes or implementing hypofractionation schemes [5, 6], their impact on environmental awareness and sustainable practice can be significant. Additionally, Roletto [7] highlighted the knowledge gap among European radiographers and RTs regarding sustainability, highlighting the urgent need to include green skills to foster a greener culture. Therefore, we would like to emphasise that empowering RTs with these skills is essential to encourage and nurture a culture of environmental responsibility within radiation therapy departments.

Kyeyune and Leech's article is an important call to action. Connecting environmental accountability with clinical practice reinforces the case for making sustainability a fundamental value in radiation oncology, and their contribution advances this critical discussion towards a more sustainable future for radiation therapy.

## Conflicts of Interest

The authors declare no conflicts of interest.

## Data Availability

Data sharing not applicable to this article as no datasets were generated or analyzed during the current study.
